# ‘Real‐world’ compensatory behaviour with low nicotine concentration e‐liquid: subjective effects and nicotine, acrolein and formaldehyde exposure

**DOI:** 10.1111/add.14271

**Published:** 2018-06-19

**Authors:** Lynne Dawkins, Sharon Cox, Maciej Goniewicz, Hayden McRobbie, Catherine Kimber, Mira Doig, Leon Kośmider

**Affiliations:** ^1^ Centre for Addictive Behaviours Research, School of Applied Sciences London South Bank University London UK; ^2^ Department of Health Behavior Roswell Park Cancer Institute Buffalo NY USA; ^3^ Barts and The London School of Medicine and Dentistry Queen Mary University of London, Wolfson Institute of Preventive Medicine London UK; ^4^ School of Psychology, College of Applied Health and Communities University of East London London UK; ^5^ ABS Laboratories Ltd, BioPark Welwyn Garden City UK; ^6^ Department of Pharmaceutics, School of Pharmacy, affiliated with the Center for the Study of Tobacco Products Virginia Commonwealth University Richmond VA USA

**Keywords:** Acrolein, compensatory behaviour, E‐cigarette, formaldehyde, nicotine, puffing patterns, subjective effects

## Abstract

**Aims:**

To compare the effects of (i) high versus low nicotine concentration e‐liquid, (ii) fixed versus adjustable power and (iii) the interaction between the two on: (a) vaping behaviour, (b) subjective effects, (c) nicotine intake and (d) exposure to acrolein and formaldehyde in e‐cigarette users vaping in their everyday setting.

**Design:**

Counterbalanced, repeated measures with four conditions: (i) low nicotine (6 mg/ml)/fixed power; (ii) low nicotine/adjustable power; (iii) high nicotine (18 mg/ml)/fixed power; and (iv) high nicotine/adjustable power.

**Setting:**

London and the South East, England.

**Participants:**

Twenty experienced e‐cigarette users (recruited between September 2016 and February 2017) vaped *ad libitum* using an eVic Supreme™ with a ‘Nautilus Aspire’ tank over 4 weeks (1 week per condition).

**Measurements:**

Puffing patterns [daily puff number (PN), puff duration (PD), interpuff interval (IPI)], ml of e‐liquid consumed, changes to power (where permitted) and subjective effects (urge to vape, nicotine withdrawal symptoms) were measured in each condition. Nicotine intake was measured via salivary cotinine. 3‐Hydroxypropylmercapturic acid (3‐HPMA), a metabolite of the toxicant acrolein, and formate, a metabolite of the carcinogen formaldehyde, were measured in urine.

**Findings:**

There was a significant nicotine concentration × power interaction for PD (*P* < 0.01). PD was longer with low nicotine/fixed power compared with (i) high nicotine/fixed power (*P* < 0.001) and (ii) low nicotine/adjustable power (*P* < 0.01). PN and liquid consumed were higher in the low versus high nicotine condition (main effect of nicotine, *P* < 0.05). Urge to vape and withdrawal symptoms were lower, and nicotine intake was higher, in the high nicotine condition (main effects of nicotine: *P* < 0.01). While acrolein levels did not differ, there was a significant nicotine × power interaction for formaldehyde (*P* < 0.05).

**Conclusions:**

Use of a lower nicotine concentration e‐liquid may be associated with compensatory behaviour (e.g. higher number and duration of puffs) and increases in negative affect, urge to vape and formaldehyde exposure.

## Introduction

Awareness and use of electronic cigarettes (e‐cigarettes) has increased rapidly during recent years, with an estimated 23.1 million smokers in the European Union (EU) reporting ever trying an e‐cigarette in 2012 1 and 12.9 million people using e‐cigarettes in the United States in 2014 2. In the United Kingdom, an estimated 2.9 million people currently use e‐cigarettes and, for the first time, in 2017 there were more ex‐smokers using e‐cigarettes (52%) than dual users (45% 3). The most commonly cited reason for use is to quit smoking, and increasing evidence suggests that e‐cigarette use may be an effective tool for this 4, 5, 6.

Nicotine delivery from e‐cigarettes varies considerably, and depends upon a variety of factors: the nicotine concentration in the e‐liquid 7, the type and power of the device and settings 8, 9, 10 as well as user characteristics, including puffing patterns 9, 11, 12. Over time, e‐cigarette users (vapers) tend to lower the nicotine concentration in their e‐liquid 13, 14. This may be due to: the belief that it is healthier; to allow changes to the device/tank (e.g. sub‐ohming—using an atomizer coil with a resistance of < 1 Ohm with increased power); or to wean off e‐cigarette/nicotine use entirely. However, a reduction in nicotine intake may not actually follow a reduction in nicotine e‐liquid concentration if users engage in compensatory puffing, such as increasing the total puff number or taking longer puffs 12, 15. In fact, in 98 vapers tested at baseline and 8 months later, while average nicotine concentration in e‐liquid decreased from 11 to 6 mg/ml, salivary cotinine levels remained stable 13. Hence, vapers may self‐titrate to a level of satisfaction which is optimal to their needs when adjusting to a lower nicotine concentration e‐liquid.

Compensatory puffing behaviour is seen in tobacco smoking 16, 17, 18, 19, with smokers achieving 60–80% of their optimal nicotine levels by engaging in this practice (i.e. by taking longer, larger volume and more frequent puffs) when switching to lower nicotine yield cigarettes 20. To date, there have only been a few studies on compensatory puffing in e‐cigarette use. Ramoa *et al*. 15 reported longer and deeper puffs in vapers using a 0‐ compared with a 36‐mg/ml nicotine e‐liquid and, in a small laboratory‐based study (*n* = 11), Dawkins *et al*. 12 observed compensatory behaviour in vapers using 6 compared with 24 mg/ml e‐liquid via more frequent and longer puffs, resulting in a doubling of e‐liquid consumed. In a very recent report, compensatory puffing (increased puff number and puff duration) was also observed when device power was set at 6 W compared with 10 W 21. Aside from puffing behaviour, vapers may engage in other forms of compensatory behaviour by adjusting the settings on the device itself. Newer‐generation devices now commonly house a control head allowing air‐flow, voltage or wattage to be adjusted. Anecdotal reports suggest that use of lower nicotine concentration e‐liquids is accompanied by a lower atomizer resistance and upward voltage or wattage adjustment. This increases the overall power output of the device which, in turn, increases aerosol production. This form of behaviour compensation, however, has received no attention in the extant empirical literature.

Compensatory behaviour, including more frequent and longer puffs and increasing power, can increase the temperature of the coil at which glycerine and propylene glycol (solvents used in e‐liquids) undergo decomposition to carbonyl compounds 22. This, in turn, may increase exposure to carcinogens such as formaldehyde, acetaldehyde and acrolein. Four studies have explored biomarker levels in human vapers’ saliva or urine, and have reported a more favourable toxicity profile than for tobacco smokers under normal usage conditions for polycyclic aromatic hydrocarbons (PAHs), tobacco‐specific nitrosamines (TSNAs) and other carcinogens, including acrolein 23. Reduced TSNAs, PAHs and volatile organic compounds (VOCs) have also been found in smokers who switched to vaping over a 2–4‐week period compared to those who continued smoking while nicotine exposure remained unchanged 24, 25. In a recent study comparing tobacco smokers with e‐cigarette‐only and nicotine replacement therapy (NRT)‐only users, carcinogens and toxicants (TSNAs, VOC) were lower in both e‐cigarette and NRT users compared with tobacco smokers 26. To date, no studies have explored biomarkers of exposure to the known human carcinogen formaldehyde or explored the effect of compensatory behaviour on carcinogen exposure.

The paucity of research on compensatory behaviour in e‐cigarette use makes it difficult to generate clear evidenced‐based health messages which can inform the public on the possible subjective, biological and toxicant effects of increased usage. Currently, the laboratory‐based studies provide some evidence of compensatory puffing, but these have used restrictive behavioural parameters which may not reflect true user behaviour. In reality, vapers are likely to adjust the power (wattage) on their devices (where device type allows) when switching to lower nicotine concentration e‐liquids according to personal preference and current needs. Vapers can adjust the power either by increasing battery output voltage or replacing the atomizer with a heating element of lower resistance (for example in ‘sub‐ohming’). In most cases, given that the atomizer resistance stays the same, by increasing voltage vapers are increasing the overall power (wattage) of the device.

In the present study, participants vaped *ad libitum* using an eVic Supreme™ with a ‘Nautilus Aspire’ tank over 4 weeks (1 week per conditions): (i) low nicotine (6 mg/ml)/fixed power; (ii) low nicotine/adjustable power; (iii) high nicotine (18 mg/ml)/fixed power; and (iv) high nicotine/adjustable power. Although it was the voltage rather than the wattage that participants adjusted, given that the atomizer resistance remained the same, increasing voltage results in an overall power increase, hence we refer to changes to power throughout. The aims were: (1) to compare the effects of nicotine concentration and power setting on: (a) puffing behaviour [puff number, puff duration, interpuff interval (IPI)]; (2) product use (e‐liquid consumed and voltage adjustment); (b) subjective effects (urge to vape, nicotine withdrawal symptoms and positive and negative effects); and (c) nicotine delivery, acrolein and formaldehyde exposure. We hypothesized significant nicotine concentration × power interactions; i.e. the lower nicotine concentration e‐liquid, especially in combination with adjustable power, would be associated with compensatory behaviour [increased puff number, longer puff duration and higher voltage (where changes to power are permitted)], reduced positive subjective effects, and increased toxicant exposure.

## Methods

### Design and ethical approval

The study was approved by London South Bank University ethics committee (UREC 1604) and conducted in accordance with the ethical standards of the 1964 Declaration of Helsinki. Participants provided written informed consent to take part and for the study to be written up for publication. A randomized, within‐participants counterbalanced design with four conditions (low nicotine/fixed power; high nicotine/fixed power; low nicotine/adjustable power; high nicotine/adjustable power) was used. Twenty‐two participants were recruited via social media and university advertising between September 2016 and February 2017. Two withdrew during week 1 but, as per protocol 27, we continued to recruit until 20 participants had completed all four conditions. Participants were asked to vape *ad libitum* using an eVic Supreme™ with a ‘Nautilus Aspire’ tank over 4 weeks (1 week per condition). The sample size was based on puff number and puff duration results from our pilot study and from Dawkins *et al*. 12, where effect sizes of between *d* = 0.74 and *d* = 0.79 were found for puff number and between *d* = 0.84 and *d* = 1.09 for puff duration. A sample of between 14 and 19 for puff number and between 11 and 17 for puff duration would allow us to detect effects at *P* < 0.05 with more than 90% power.

### Procedure

The published protocol describes the procedure and measures in detail 27. Participants were eligible to participate if they were: aged 18+; experienced and exclusive daily e‐cigarette users (daily use for > 3 months); currently using a second‐ or third‐generation e‐cigarette; using ≥ 12 mg/ml nicotine concentration e‐liquid (to ensure participants were familiar with higher levels of nicotine) or sub‐ohming (any nicotine level); non‐smokers as confirmed by carbon monoxide (CO) levels in expired breath of ≤ 10 parts per million (p.p.m.) We excluded pregnant or lactating females, current smokers or users of marijuana or other illicit drugs and those with a serious medical condition (self‐reported). Participants met with the researcher on five separate occasions (at baseline and the end of each of the four experimental conditions). At baseline participants provided written informed consent, demographic characteristics and smoking/vaping history then sampled four e‐liquids (tobacco, fruit, bakery and menthol), selecting one to be used for the next 4 weeks. Participants were provided with an eVic Supreme™ by Joytech fitted with a ‘Nautilus Aspire’ tank housing a BVC atomizer (1.6 Ohm) and seven 10‐ml bottles of e‐liquid for the week (6 or 18 mg/ml according to condition).

For each puff, the eVic records: time of the puff, puff length (in seconds), atomizer resistance, voltage and wattage. To ensure device familiarity before changes were permitted, the first 2 weeks were always fixed at 4.0 V (10 W) with the widest air‐flow setting on the tank. Changes to voltage and airflow were permitted during the last 2 weeks. Participants could adjust the airflow by turning a horizontal dial on the tank. However, no changes to airflow were made, so this is not reported further. Voltage could be adjusted (between 3.0 and 6.0 V) by turning a dial under the display unit on the eVic. Given that the atomizer resistance was fixed at 1.6 Ohm, adjusting the voltage upwards results in increased wattage (overall power output). Participants were assigned randomly to start on either 6 or 18 mg/ml nicotine concentration e‐liquid, giving rise to four possible orders. Participants were instructed to refrain from using their own devices and e‐liquids for the duration of the study.

At baseline and each follow‐up time‐point, breath CO samples were taken (to confirm that participants had not smoked recently) and saliva and urine were provided and sent to Advanced Bioanalytical Service (ABS) Laboratories Ltd (Welwyn Garden City, UK), where they were frozen at −20° until assayed. Saliva was assayed for cotinine 28 to determine nicotine intake. Urine was assayed for 3‐HPMA to estimate acrolein exposure 24 and formate for formaldehyde exposure 29. Both were adjusted for creatinine concentration.

At all five visits, participants reported their urge to vape, withdrawal symptoms and positive and negative effects during the last week. At the end of each condition, puffing and power data were downloaded from the device using myVapor™ software. Volume of e‐liquid used was recorded based on participant self‐report. At the end of the study participants were debriefed, thanked for their participation and compensated £60 for their time and travel.

### Measures

Demographic and smoking‐ and vaping‐related information was collected at baseline, including: length of time since quitting smoking; e‐cigarette device used; estimated daily volume of e‐liquid consumed; nicotine concentrations used; self‐rated addiction to e‐cigarettes (0–100%); and e‐cigarette dependence (measured using the Penn State E‐cigarette Dependence Index) 30.

At all five visits, participants completed a modified version of the Mood and Physical Symptoms Scale 31 for urge to vape and nicotine withdrawal symptoms. Time spent with urges during the past week was rated from 0 = not at all to 5 = all the time; strength of urges were rated from 0 = no urges to 5 = extremely strong and withdrawal symptoms were rated from 1 = not at all to 5 = extremely. Positive (e.g. hit, satisfaction) and negative (nausea, headache) effects were rated on visual analogue scales (VAS) ranging from 0–100% 11.

### Data analysis

#### Puffing topography and user behaviour

Data from the first/last day of each condition were excluded from analysis as (a) user behaviour on the first day is likely to reflect familiarity and adjustment to the device; and (b) these represented condition change‐over days. Puffing data were screened and all button presses < 1 second (false button presses or non‐starts) were deleted. Puffing data from one participant were lost due to a problem with the device. The mean number of days per condition was 6, but due to appointment re‐arrangements, occasional use of own device or days of non‐use (due to flights, hospital admission), this ranged from 4 to 13. Averages (mean for voltage, daily puff number, puff duration and liquid consumption and median for IPI) for each condition were computed by summing the data points for that variable and dividing by the number of compliant days in that condition.

Data were analysed using SPSS version 21. Distributions for most variables were normal, although withdrawal symptoms, negative effects, 3‐HPMA and formate were positively skewed. Repeated‐measures analyses of variance (ANOVA) with nicotine concentration (6 versus 18 mg/ml) and power type [fixed (F) versus adjustable (A)] were conducted for each variable (except voltage, which was fixed in two of four conditions). Order of testing was added as an additional between‐subjects variable, but is mentioned only where a significant main effect or interaction was found. The accepted alpha level was *P* < 0.05. Where significant nicotine × power interactions were found, *post‐hoc* paired‐sample *t*‐tests were conducted (for 6F versus 6A; 18F versus 18A; 6F versus 18A and 6A versus 18A, as appropriate). The alpha level for *post‐hoc* tests was subjected to a Bonferroni adjustment (0.05/4); the accepted alpha level was therefore 0.01. ANOVA results for the interaction and main effects of nicotine concentration and power are displayed in Table 2. *Post‐hoc* test statistics and any order effects are included in the text below.

## Results

Participants’ characteristics are shown in Table 1. On average, participants had quit smoking for 26 months, had a mean score of 12.05 on the Penn State E‐cigarette Dependence Index and used a mean of 6.57 ml of e‐liquid a day. Baseline salivary cotinine levels ranged from 39 to 719 ng/ml.

**Table 1:** Participant demographics and baseline characteristics.

	n	Percentage	Mean	SD	Min.	Max.
Gender						
Male	12	60				
Female	8	40				
Age (years)	20		37.90	10.66	23	62
Ethnicity						
White	19	95				
Mixed‐race	1	5				
Qualification						
GSCE‐levels	10	50				
A‐levels	5	25				
Undergraduate level (5–6)	2	10				
Post‐graduate level (7 and above)	3	15				
Occupational status						
Employed	14	70				
Retired	1	5				
Self‐employed	5	25				
Length of time quit smoking (months)	20		25.95	23.35	3	108
E‐cigarette addiction (0–100%)	20		70.15	21.90	40	100
Penn State E‐cigarette Dependence Index	20		12.05	4.02	4	20
Baseline cotinine levels (ng/ml)	20		324	219	39	719
Baseline CO levels	19		3.90	2.77	0	9
Daily liquid volume consumed (ml)	16		6.57	3.10	1.3	10
Estimated puffs per day	6		180	80	80	300
Current model most used						
Rechargeable non‐cigalike (2nd generation)	12	60				
Modular systems (including sub‐ohms)	8	40				
Main nicotine concentration used						
Non‐sub‐ohmers	5	25	14.13	4.16	6	24
Sub‐ohmers (incl daily & occasional use)	15	75	8.40	4.93	3	15

CO = carbon monoxide; GCSE = General Certificate of Secondary Education; SD = standard deviation.

### Puffing topography

Puffing topography data for each condition are presented in Table 2 (individual puffing patterns are available in the Supporting information).

**Table 2:** Mean puffing patterns, product use, subjective effects and biomarker levels across the four conditions.

	6 mg/ml fixed power		18 mg/ml fixed power		6 mg/ml adjustable power		18 mg/ml adjustable power		Nicotine	Power	Nicotine × power
	Mean	SD	Mean	SD	Mean	SD	Mean	SD	F (P)	F (P)	F (P)
Puffing topography											
Daily puff number	338	161	279	127	308	135	272	128	16.39 (0.001)	2.16 (0.16)	0.98 (0.34)
Puff duration	4.46	1.22	3.61	0.97	3.81	1.11	3.91	1.44	18.30 (0.001)	1.69 (0.21)	12.12 (0.003)
Interpuff interval	34.22	20.08	41.22	26.23	39.32	26.80	37.32	27.18	0.33 (0.58)	0.04 (0.86)	5.76 (0.03)
Product use											
Daily (ml) liquid consumed	6.19	3.74	4.63	2.13	5.79	3.63	4.79	2.35	6.25 (0.02)	0.07 (0.80)	0.75 (0.40)
Voltage (wattage)	4 (10)	–	4 (10)	–	4.5 (12.66)	0.7	4.3 (11.56)	0.8	–	–	–
Subjective effects											
Urge to vape	3.20	1.11	2.40	0.10	3.00	0.86	2.45	0.83	16.74 (0.001)	0.77 (0.39)	1.95 (0.18)
Strength of urges	2.90	1.17	2.15	0.67	2.85	1.18	2.45	1.10	14.63 (0.001)	0.91 (0.36)	3.56 (0.08)
Withdrawal symptoms	9.05	4.56	7.80	2.59	7.45	2.91	6.80	1.36	4.88 (0.04)	8.78 (0.01)	2.51 (0.13)
Positive effects	50.71	19.82	62.64	20.30	63.85	21.58	63.42	22.25	2.06 (0.17)	4.69 (0.05)	26.48 (0.001)
Negative effects	15.05	19.49	22.25	22.75	11.26	9.66	14.74	12.84	3.37 (0.09)	5.36 (0.03)	0.38 (0.55)
Biomarkers											
Salivary cotinine (ng/ml)	250.45	188.23	402.52	190.00	274.95	172.81	405.21	192.80	17.49 (0.001)	0.15 (0.70)	0.12 (0.73)
3‐HPMA (ng/mg creatinine)	211.83	133.09	262.73	202.50	224.13	343.39	378.35	467.35	0.82 (0.38)	0.29 (0.60)	0.09 (0.77)
Formate (μg/mg creatinine)	10.52	8.00	9.62	7.28	18.01	23.56	7.61	7.24	4.58 (0.05)	1.88 (0.19)	6.92 (0.02)

SD = standard deviation; 3‐HPMA = 3‐hydroxypropylmercapturic acid.

#### Puff number

The nicotine × power interaction was not statistically significant, but there was a main effect of nicotine with a higher puff number in the 6 versus 18 mg/ml nicotine condition.

#### Puff duration

There was a significant nicotine concentration × power interaction. Puff duration was significantly longer with 6 compared with 18 mg/ml nicotine e‐liquid in the fixed power condition [6F versus 18F: *t*_18_ = 5.26, *P* = 0.000, mean difference = 0.85, 95% confidence interval (CI) = – 0.51 to 1.19) and longer with 6 fixed versus 6 adjustable power (*t*_18_ = 3.15, *P* = 0.006, mean difference = 0.66, 95% CI = 0.22–1.09). There was also a significant setting × order interaction (*F*_3,15_ = 4.61, *P* = 0.02, η_p_^2^ = 0.48), with those starting and finishing on 6 mg/ml demonstrating a shorter puff duration when settings were adjustable.

#### IPI

IPI was calculated from information on puff timings in each condition. The median, rather than mean, IPI was taken due to the highly skewed data which included long periods of inactivity/non‐use (due presumably to sleeping or working where vaping is not permitted). IPI could not be calculated for some conditions for two participants due to corrupted data files which distorted puff times.

The nicotine × power interaction was statistically significant. Inspection of Table 2 reveals that IPI was shortest under the 6 mg/ml fixed condition and was similar under all other conditions. These differences however, fell short of statistical significance in *post‐hoc* tests.

### Product use

#### E‐liquid consumption

There was no nicotine × power interaction, although a significantly greater volume (ml) of e‐liquid per day was consumed in the 6 versus 18 mg/ml nicotine condition (main effect of nicotine).

#### Changes to power setting (voltage)

When participants were permitted to adjust the power, compared to the fixed 4 V (10 W) condition, 13 increased the voltage, two made no changes and four decreased it in the 6 mg/ml nicotine condition. In the 18 mg/ml nicotine condition, six increased the voltage, five made no changes and seven decreased it. Overall, mean voltage was higher in the adjustable [mean = 4.39, standard deviation (SD) = 0.75] compared with the fixed (4 V) power condition; participants increased the voltage by a mean of 0.5 v (95% CI = 0.17–0.84) in the low nicotine condition and 0.3 v (95% CI = 0.12–0.65) in the high condition.

### Subjective effects

Mean scores for subjective effects in each condition are presented in Table 2.

#### Urge to vape

Although there was no significant nicotine concentration × power interaction for either urge to vape or strength of urges, both were significantly higher in the 6 compared with the 18 mg/ml nicotine condition (main effect of nicotine).

#### Withdrawal symptoms

The nicotine × power interaction was not statistically significant, although nicotine withdrawal symptoms were higher in the 6 versus 18 mg/ml condition (main effect of nicotine) and in the fixed versus adjustable power condition (main effect of power).

#### Positive effects

There was a significant nicotine × power interaction for positive effects. *Post‐hoc* tests revealed that positive effects were lower in the 6 versus the 18 mg/ml nicotine condition under fixed power (6F versus 18F: *t*_19_ = −2.96, *P* = 0.008, mean difference = −11.93, 95% CI = −20.38 to −3.49) and lower under 6F compared with 6A (*t*_19_ = −3.74, *P* = 0.001, mean difference = −13.14, 95% CI = −20.49 to −5.79).

#### Negative effects

Self‐reported adverse effects were very low across conditions and there was no significant nicotine × power interaction. There was a significant main effect of power, with higher ratings of adverse effects in the fixed versus the adjustable power condition.

### Biomarkers analysis

Results of the biomarker analyses are presented in Table 2.

#### Nicotine delivery (salivary cotinine)

The interaction between nicotine concentration and power was not statistically significant, but there was a main effect of nicotine with higher salivary cotinine levels in the 18 mg/ml compared with the 6 mg/ml nicotine condition. There was also a significant main effect of order, with those receiving 18 mg/ml first achieving higher overall cotinine levels (*F*_3,15_ = 6.54, *P* = 0.005, η_p_^2^ = 0.57).

#### Acrolein (3‐HMPA) and formaldehyde (formate) exposure

Four urine samples were not received for analysis. For 3‐HPMA there was no significant nicotine × power interaction or main effects. There was a significant nicotine × power interaction for formate; levels were higher in the 6A condition compared with all other conditions, although these differences were not statistically significant in *post‐hoc t*‐tests. As the 3‐HPMA and formate variables were positively skewed by a few extreme high scores, the analysis was repeated with outliers removed (six in each case). The results remained unchanged for 3‐HPMA. For formate the interaction remained significant (*F*_1,12_ = 13.33, *P* = 0.003, η_p_^2^ = 0.53) and *post‐hoc* tests revealed a significant difference between 6A and 18A (*t*_12_ = 3.16, *P* = 0.008, mean difference = 4.93, 95% CI = 1.53–8.33), although the 6A versus 6F difference fell short of the adjusted level of significance (*t*_12_ = −2.41, *P* = 0.03, mean difference = −2.77, 95% CI = −5.27 to –0.27).

## Discussion

Our study is the first to document real‐world compensatory behaviour (puffing patterns and changes to power) with low nicotine concentration e‐liquid and to explore the effects on nicotine intake and toxicant/carcinogen exposure. Consistent with our hypothesis and earlier laboratory study 12, participants increased their puff number and puff duration, decreased their IPI and consumed more e‐liquid in the low (6 mg/ml) compared with the high (18 mg/ml) nicotine condition. The effect of nicotine on puff duration was more pronounced when power settings were fixed. Despite this evidence of compensatory behaviour, nicotine intake (measured via salivary cotinine) remained higher in the high nicotine condition. Urge to vape and nicotine withdrawal symptoms were higher and positive effects were lower in the low nicotine condition, particularly when the power was fixed. When changes to power settings were permitted, participants increased the voltage to a greater extent in the low compared with the high nicotine condition. While acrolein levels did not differ across conditions, formaldehyde exposure was higher in the low nicotine, adjustable power condition. Overall our findings add to the evidence base supporting compensatory behaviour with lower nicotine concentration e‐liquid, which results in reduced positive subjective effects and may increase formaldehyde exposure.

Our puffing topography findings are consistent with our earlier laboratory‐based study which also found increased puff number and puff duration with a lower nicotine concentration e‐liquid 12, supporting the notion that, as with tobacco smokers, vapers engage in compensatory puffing in an attempt to self‐titrate with a lower nicotine concentration e‐liquid. We also permitted changes to power settings to reflect how experienced vapers using newer‐generation devices behave in real‐world conditions. Participants were more likely to increase the power in the low nicotine condition, resulting in a shorter puff duration (but no change to puff number) compared with fixed power settings. Nevertheless, nicotine intake remained higher in the high nicotine condition regardless of whether or not power was fixed, suggesting that compensatory puffing and changes to power were not adequate to raise nicotine intake to the level achieved via a high (18 mg/ml) nicotine e‐liquid concentration. In fact, baseline salivary cotinine levels fell roughly mid‐point between the levels achieved in the high and low nicotine conditions suggesting that, as with tobacco smoking and with vapers in our earlier study, upwards and downwards self‐titration is incomplete 12, 16, 32.

In relation to subjective effects, urge to vape, strength of urges and withdrawal symptoms were higher, and positive effects were lower, in the low nicotine condition. Although urge to vape was unaffected by changes to power‐settings, withdrawal symptoms and positive effects were improved, suggesting that increasing the power to the battery can improve the subjective experience when using a lower nicotine concentration e‐liquid. Nevertheless, although the device used here, as with many newer‐generation devices, allows adjustment to the power, many standard cigalike and second‐generation devices do not. Our sample were experienced vapers, many of whom reported sub‐ohming (of the whole sample: 40% daily; 75% occasionally) and were therefore familiar with changing device settings. In the absence of knowledge or mechanisms to adjust power, our findings suggest that a lower nicotine concentration e‐liquid is associated with higher urges and withdrawal symptoms and reduced overall satisfaction.

Levels of 3‐HPMA did not differ across nicotine or power conditions and were within the range found in exclusive e‐cigarette users in other studies 23, 25, although slightly higher than those reported by Shahab *et al*. 26. To our knowledge, we are the first to measure formate as an estimate of formaldehyde exposure, a known human carcinogen 33 in e‐cigarette users. Levels of formate were higher in the low versus the high nicotine condition, particularly when users were permitted to adjust the power. These findings are consistent with our previous report of increased formaldehyde in e‐cigarette aerosol generated using a more intensive puffing regimen 34. Although these results are suggestive of an effect of compensatory behaviour on formaldehyde exposure, they are by no means conclusive; the sample size was small and the influence of other foods and drugs (not measured here) known to influence formaldehyde exposure cannot be ruled out.

Our study has several limitations. Although participants were not aware of the study aims, they were not blinded to the nicotine e‐liquid concentration which may have influenced their puffing patterns and subjective reporting. In terms of compliance, reports of using non‐study devices was reported occasionally and days of non‐use occurred (e.g. during a flight or hospital admission). Although these days were removed from the puffing analysis, this could have influenced the biomarkers analysis. Occasional smoking (including marijuana or hookah use) may also have occurred; although CO levels were all below 10 p.p.m., several were between 6 and 9 p.p.m. and we did not have the resources to confirm lack of marijuana use biochemically. However, cross‐referencing these higher CO values against nicotine, 3‐HPMA and formate results did not reveal higher levels compared with the rest of the sample. Our participants were all experienced e‐cigarette users, had vaped on average for 2 years and 40% were daily sub‐ohmers. The puffing patterns and behaviour of these users may not therefore reflect the typical vaper or smokers who have recently transitioned to vaping. Finally, vapers are unlikely to transition directly from a very high (18 mg/ml) to a very low (6 mg/ml) nicotine concentration and in practice may move through an intermediate stage (12 mg/ml). Whether smaller changes in nicotine concentrations are associated with changes to puffing topography and subjective effects is unknown.

In conclusion, use of a lower nicotine concentration e‐liquid is associated with compensatory puffing, reduced subjective effects and, where permitted, increases to the power of the device. Our findings suggest that this compensatory behaviour is not sufficient to fully compensate for lower nicotine delivery and may be associated with increased formaldehyde exposure. Switching to a lower nicotine concentration e‐liquid may therefore be unsatisfying, triggering compensatory behaviour which increases e‐liquid consumption and may increase health risks. Although our formaldehyde findings require replication, our data suggest that vapers should carefully consider switching to lower nicotine concentration e‐liquids.

## Declaration of interests

This study was support by grant C50878/A21130 from Cancer Research UK. L.D. has conducted research for independent electronic cigarette companies. These companies had no input into the design, conduct or write up of the projects. She has also acted as a consultant for the pharmaceutical industry and as an expert witness in a patent infringement case (2015). M.G. received a research grant from Pfizer and serves on an advisory board to Johnson & Johnson, manufacturers of smoking cessation medications. H.M. has received honoraria for speaking at research symposia and received benefits in kind and travel support from, and has provided consultancy to, the manufacturers of smoking cessation medications. L.K. is supported by the National Institute on Drug Abuse of the National Institutes of Health under Award Number P50DA036105 and the Center for Tobacco Products of the US Food and Drug Administration. The content is solely the responsibility of the authors and does not necessarily represent the views of the NIH or the FDA. L.K. was also an employee of the Institute of Occupational Medicine and Environmental Health. One of the institute's objectives is outsourcing for the industrial sector, including manufacturers of e‐cigarettes. S.C., C.K. and M.D. have no conflicts of interest to declare.

## Supporting information


**Table S1** Individual puffing patterns and group averages for each condition.
