# Supplementary material for: ‘Real‐world’ compensatory behaviour with low nicotine concentration e‐liquid: subjective effects and nicotine, acrolein and formaldehyde exposure
Source: Addiction. 2018 Jun 19;113(10):1874–82. doi: 10.1111/add.14271 (PMC6150437; doi:10.1111/add.14271)
Supplement: Supplementary file 1 — Table S1 Individual puffing patterns and group averages for each condition. [file ADD-113-1874-s001.docx]

|  | **6mg/mL fixed power setting** | | | | | | **18mg/mL fixed power settings** | | | | | | **6mg/mL adjustable power settings** | | | | | | **18mg/mL adjustable power settings** | | | | | | |
| --- | --- | --- | --- | --- | --- | --- | --- | --- | --- | --- | --- | --- | --- | --- | --- | --- | --- | --- | --- | --- | --- | --- | --- | --- | --- |
| P | PN | PD | IPI | liquid | V | W | PN | PD | IPI | liquid | V | W | PN | PD | IPI | liquid | V | W | PN | PD | IPI | liquid | V | W |  |
| 1 | 398 | 5.13 | 32 | 5.83 | 4 | 10 | 268 | 3.54 | 31 | 4.17 | 4 | 10 | 445 | 3.62 | 31 | 5.00 | 5.1 | 16.26 | 276 | 2.91 | 46 | 3.33 | 5.2 | 16.90 |  |
| 2 | 123 | 3.04 | 38 | 1.17 | 4 | 10 | 136 | 2.95 | 51 | 2.50 | 4 | 10 | 116 | 3.29 | 34 | .83 | 4 | 10 | 155 | 3.47 | 38 | 3.33 | 3.9 | 9.51 |  |
| 3 | 158 | 5 | 55 | 2.50 | 4 | 10 | 134 | 3.48 | 53 | 2.40 | 4 | 10 | 216 | 3.96 | 41 | 4.50 | 4.2 | 11.03 | 180 | 4.74 | 40 | 2.50 | 3.8 | 9.03 |  |
| 4 | 692 | 4.81 | 16 | 11.67 | 4 | 10 | 585 | 3.27 | 14 | 7.50 | 4 | 10 | 500 | 4.72 | 20 | 15.00 | 4.3 | 11.56 | 506 | 5.17 | 21 | 5.83 | 3.4 | 7.23 |  |
| 5 | 464 | 4.41 | 44 | 5.83 | 4 | 10 | 405 | 3.49 | 54 | 2.83 | 4 | 10 | 357 | 3.5 | 83 | 4.17 | 4.4 | 12.10 | 429 | 2.81 | 56 | 3.08 | 4.4 | 12.10 |  |
| 6 | 149 | 2.56 | 7 | 3.57 | 4 | 10 | 128 | 2.03 |  | 3.00 | 4 | 10 | 121 | 3 | 9 | 4.17 | 3.7 | 8.56 | 103 | 2.57 | 7 | 3.60 | 4 | 10 |  |
| 7 | 137 | 2.35 | 8 | 2.92 | 4 | 10 | 114 | 2.04 | 10 | 4.00 | 4 | 10 | 115 | 2.47 | 10 | .83 | 3.7 | 8.56 | 101 | 2.41 | 7 | 1.60 | 4 | 10 |  |
| 8 | 180 | 4.75 | 24 | 4.00 | 4 | 10 | 121 | 4.26 | 32 | 2.50 | 4 | 10 | 174 | 2.57 | 22 | 3.57 | 5.5 | 18.91 | 195 | 2.38 | 33 | 6.25 | 5.3 | 17.56 |  |
| 9 | 362 | 6.65 | 53 | 10.00 | 4 | 10 | 329 | 5.08 | 77 | 8.00 | 4 | 10 | 298 | 6.64 | 42 | 8.33 | 4.3 | 11.56 | 260 | 7.14 | 42 | 10.00 | 3.4 | 7.23 |  |
| 10 | 439 | 4.63 | 77 | 8.33 | 4 | 10 | 292 | 4 | 111 | 5.83 | 4 | 10 | 396 | 4.73 | 88 | 10.00 | 4.2 | 11.03 | 301 | 3.98 | 94 | 3.33 | 4 | 10 |  |
| 11 | 474 | 4.45 | 43 | 5.00 | 4 | 10 | 328 | 4.98 | 47 | 5.00 | 4 | 10 | 346 | 3.37 | 58 | 4.17 | 5.7 | 20.31 | 279 | 3.25 | 64 | 4.50 | 6 | 22.50 |  |
| 12 | 284 | 3.23 | 8 | 3.00 | 4 | 10 | 217 | 2.97 | 8 | 2.50 | 4 | 10 | 243 | 3.37 | 9 | 3.33 | 4 | 10 | 192 | 2.99 | 9 | 2.50 | 4 | 10 |  |
| 13 |  |  |  | 6.67 |  |  |  |  |  | 5.83 |  |  |  |  |  | 7.50 |  |  |  |  |  | 7.50 |  |  |  |
| 14 | 551 | 5.76 | 21 | 14.17 | 4 | 10 | 377 | 5.04 | 31 | 9.29 | 4 | 10 | 503 | 5.7 | 25 | 10.00 | 4.3 | 11.56 | 475 | 6.1 | 26 | 5.00 | 3.6 | 8.10 |  |
| 15 | 269 | 5.64 | 17 | 10.00 | 4 | 10 | 246 | 5.13 | 22 | 6.67 | 4 | 10 | 214 | 3.01 | 13 | 8.33 | 6 | 22.50 | 208 | 2.48 | 12 | 8.33 | 6 | 22.50 |  |
| 16 | 194 | 5.56 | 48 | 3.89 | 4 | 10 | 190 | 3.56 | 27 | 2.50 | 4 | 10 | 181 | 4.32 | 74 | 1.92 | 4.5 | 12.66 | 185 | 5.78 | 53 | 4.17 | 3.6 | 8.10 |  |
| 17 | 344 | 3.3 | 20 | 3.75 | 4 | 10 | 434 | 2.33 | 21 | 3.33 | 4 | 10 | 393 | 2.15 | 18 | 7.00 | 5.5 | 18.91 | 341 | 2.54 | 19 | 4.50 | 4.5 | 12.66 |  |
| 18 | 531 | 6.04 | 26 | 12.50 | 4 | 10 | 381 | 3.96 | 29 | 6.00 | 4 | 10 | 519 | 4.38 | 28 | 3.50 | 4.4 | 12.10 | 504 | 5 | 17 | 9.00 | 3.6 | 8.10 |  |
| 19 | 300 | 3.7 | 63 | 6.67 | 4 | 10 | 312 | 3.56 | 60 | 5.83 | 4 | 10 | 359 | 3.75 | 63 | 10.00 | 3.9 | 9.51 | 182 | 4.99 | 24 | 5.00 | 4.3 | 11.56 |  |
| 20 | 364 | 3.75 | 46 | 2.40 | 4 | 10 | 307 | 2.97 | 64 | 2.86 | 4 | 10 | 364 | 3.76 | 79 | 3.60 | 3.9 | 9.51 | 307 | 3.61 | 101 | 2.50 | 4 | 10 |  |
|  |  |  |  |  |  |  |  |  |  |  |  |  |  |  |  |  |  |  |  |  |  |  |  |  |  |
| Group mean (SD) | 338 (161) | 4.46  (1.22) | 34 (20) | 6.19  (3.74) | 4 | 10 | 279 (127) | 3.61  (0.97) | 41 (26) | 4.63  (2.13) | 4 | 10 | 308 (135) | 3.81  (1.11) | 39  (27) | 5.79  (3.63) | 4.5  (0.7) | 12.66  (4.23) | 272  (128) | 3.91  (1.44) | 37 (27) | 4.79  (2.35) | 4.3  (0.8) | 11.56  (4.72) |  |

**Supplementary Table:** *Individual puffing patterns and group averages for each condition*

*P = participant; PN = Puff Number (daily mean); PD = Puff Duration (in seconds); IPI = Inter-puff-interval (median, in seconds); liquid = mean daily liquid consumed (in mL);V = Voltage; W = Wattage*
